# GWAS identifies candidate susceptibility loci and microRNA biomarkers for acute encephalopathy with biphasic seizures and late reduced diffusion

**DOI:** 10.1038/s41598-021-04576-y

**Published:** 2022-01-25

**Authors:** Mariko Kasai, Yosuke Omae, Yosuke Kawai, Akiko Shibata, Ai Hoshino, Masashi Mizuguchi, Katsushi Tokunaga

**Affiliations:** 1 Department of Developmental Medical Sciences, Graduate School of Medicine, The University of Tokyo, School of Medicine Bldg. 3, Rm. N205, 7-3-1 Hongo Bunkyo-ku, Tokyo, 113-0033 Japan; 2 Department of Pediatrics, Graduate School of Medicine, The University of Tokyo, Tokyo, Japan; 3 Genome Medical Science Project, National Center for Global Health and Medicine, Tokyo, Japan

**Keywords:** Genetics, Neurology

## Abstract

Acute encephalopathy with biphasic seizures and late reduced diffusion (AESD) is a severe encephalopathy preceded by viral infections with high fever. AESD is a multifactorial disease, however, few disease susceptibility genes have previously been identified. Here, we conducted a genome-wide association study (GWAS) and assessed functional variants in non-coding regions to study genetic susceptibility in AESD using 254 Japanese children with AESD and 799 adult healthy controls. We also performed a microRNA enrichment analysis using GWAS statistics to search for candidate biomarkers in AESD. The variant with the lowest *p*-value, rs1850440, was located in the intron of serine/threonine kinase 39 gene (*STK39*) on chromosome 2q24.3 (*p* = 2.44 × 10^−7^, odds ratio = 1.71). The minor allele T of rs1850440 correlated with the stronger expression of *STK39* in peripheral blood. This variant possessed enhancer histone modification marks in *STK39*, the encoded protein of which activates the p38 mitogen-activated protein kinase (MAPK) pathway. In the replication study, the odds ratios of three SNPs, including rs1850440, showed the same direction of association with that in the discovery stage GWAS. One of the candidate microRNAs identified by the microRNA enrichment analysis was associated with inflammatory responses regulated by the MAPK pathway. This study identified *STK39* as a novel susceptibility locus of AESD, found microRNAs as potential biomarkers, and implicated immune responses and the MAPK cascade in its pathogenesis.

Acute encephalopathy with biphasic seizures and late reduced diffusion (AESD) is a syndrome of severe acute encephalopathy predominantly affecting infants and small children. Preceded by high fever due to common viral infections, AESD is characterized by a biphasic clinical course^1^. The early phase typically begins with febrile convulsive status epilepticus, followed by post-ictal coma. At this stage, cranial MRI findings are normal, and there is currently no biomarker to differentiate AESD from prolonged febrile seizures. After 3–7 days of waking, the late phase is manifested by a cluster of focal seizures, followed by a second coma. Cranial MRI in the late phase shows cerebral cortical lesions of reduced diffusion, indicating cellular edema of the subcortical white matter^2^. After the recovery of consciousness, various signs of cerebral cortical dysfunction become apparent. Two thirds of patients are left with neurologic sequelae^3,4^. Current treatment of AESD remains largely symptomatic. Immediate diagnosis of AESD based on its new biomarkers is needed because early treatments, such as targeted temperature management, could prevent development of AESD^5^. AESD is an important cause of postnatal brain damage and neurological handicaps in Japanese children despite its low incidence of 100–200 cases per year^3^. No gender difference has been noted^3,4^. The number of AESD cases is markedly larger in Japan than in the rest of the world, suggesting genetic susceptibility in Japanese individuals. However, whole-genome approaches have never been applied to identify genetic variants associated with AESD.

MicroRNAs (miRNAs) are short noncoding RNAs that post-transcriptionally regulate gene expression^6,7^. Some miRNAs serve as disease biomarkers because their expression is caused by biological reactions, such as cellular stress and inflammation^8^. An analysis using genome-wide association study (GWAS) statistics was recently developed to detect genetic relationships between miRNAs and their target gene pairs^9^.

We herein conducted GWAS on AESD in Japanese individuals to identify novel susceptibility loci and clarify the genetic architecture of this disease. We also performed a miRNA enrichment analysis using GWAS data to detect candidate biomarkers and elucidate its pathogenesis in more detail.

## Results

### Whole-genome imputation and GWAS to identify susceptibility loci associated with AESD

**Figure 1:**
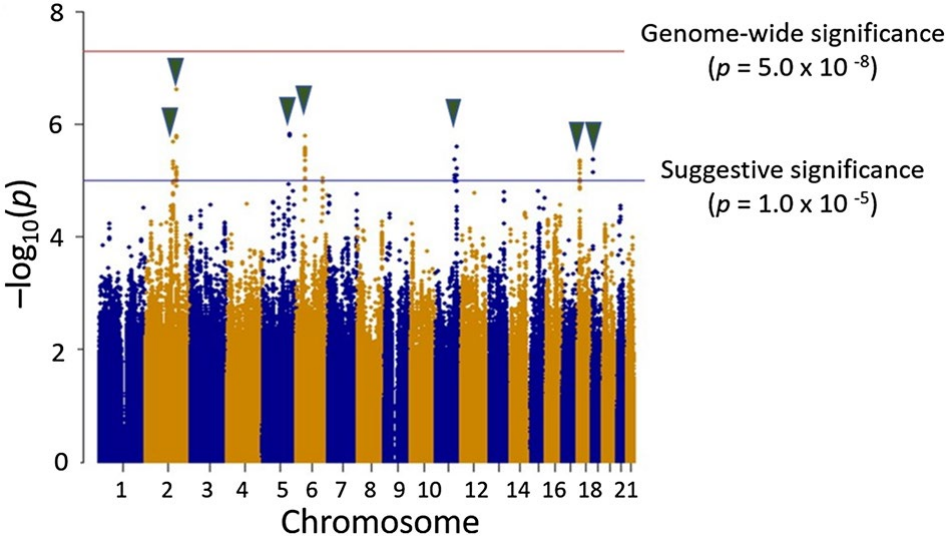
Manhattan plot of AESD GWAS after whole-genome imputation. The horizontal red and blue lines show genome-wide significance (*p* < 5.0 × 10^−8^) and suggestive significance level (*p* < 1.0 × 10^−5^), respectively.

**Figure 2:**
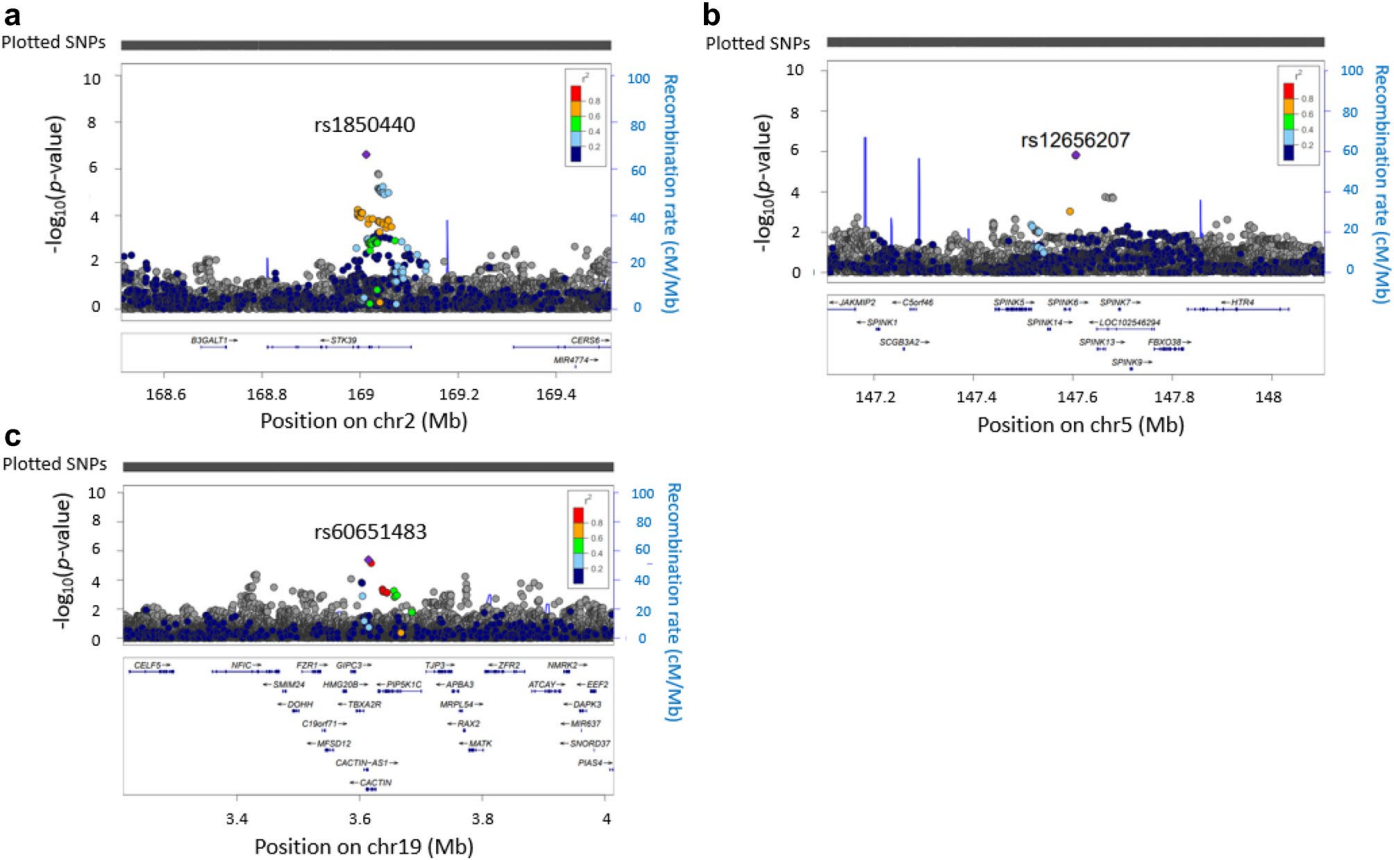
Regional plots for 3 variants with replicable odds ratios. Regional plots of susceptibility loci based on an association analysis in the GWAS. The purple dots indicate the focused variants. (**a**) Regional plot for rs1850440, (**b**) regional plot for rs12656207 and (**c**) regional plot for rs60651483.

We collected 254 Japanese pediatric cases of AESD and 799 healthy adult controls after genotyping the samples in the present study (see “Materials and Methods” for details). The clinical characteristics of the cases are shown in Table 1. Whole-genome imputation was conducted using a phased reference panel of 2,049 Japanese individuals (2KJPN panel)^10,11^ and our genotype data to perform high-density association mapping for susceptibility loci with AESD. After whole-genome imputation and quality control (QC) procedures, 252 patients and 792 controls with 3,289,568 autosomal SNPs and short INDELs remained for subsequent analyses. Seven novel loci achieved the suggestive significance associated with AESD (Fig. 1). No SNP reached the genome-wide significance level after whole-genome imputation. SNPs with *p*-values less than 1.0 × 10^−5^ were listed in Supplementary Data. The strongest associated SNP, rs1850440, located in the intron of the serine/threonine kinase 39 gene (*STK39*) on chromosome 2q24.3, was suggestively associated with AESD (*p* = 2.44 × 10^−7^, odds ratio = 1.71) (Fig. 2a). Furthermore, we found another SNP on the same chromosome 2q24.3, rs12692878 showing the suggestive significance level of a protective association with AESD (*p* = 7.57 × 10^−6^, odds ratio = 0.63). Therefore, we performed a conditional analysis conditioning on rs1850440 to confirm whether there was a secondary association that was independent of rs1850440. There was no secondary signal in the 2q24.3 region after the conditional analysis (Supplementary Fig. S1).

**Table 1:** Summary of clinical characteristics of patients with AESD in GWAS.

Clinical characteristics	Number of the patients	
	N = 254	%
**Sex**		
Male	121	47.6
Female	133	52.4
**Age (months)**		
< 12	51	20
12–24	110	43.3
> 24	93	36.6
**Early seizures**^**a**^		
Status epilepticus (> = 15 min)	212	83.5
Short seizure (< 15 min)	40	15.7
Unknown	2	0.8
**Late seizures**^**b**^		
Yes	199	78.3
No	46	18.1
Unknown	9	3.5
**MRI findings**^**c**^		
Yes	242	95.3
No	12	4.7
**Outcome**		
Full recovery	42	16.5
Neurological sequelae^d^	170	66.9
Death	0	0
Unknown	42	16.5

^a^Early seizures: generalized convulsion in the early phase of AESD.

^b^Late seizures: cluster of focal seizures in the late phase.

^c^MRI findings: characteristic lesions in the cerebral subcortical white matter detected by cranial MRI.

^d^Neurological sequelae: intellectual and/or motor disability.

### Validation and replication test of candidate SNPs associated with AESD

To validate the associations of the SNPs identified by the GWAS, we performed association tests of the 8 candidate SNPs in the same 252 AESD patients as in the GWAS using TaqMan genotyping assays (Thermo Fisher Scientific, Inc.). We selected 7 loci with SNPs with *p* < 1.0 × 10^−5^ in GWAS for validation. Of these variants, 7 SNPs were selected as proxies in the strong linkage disequilibrium (LD) of each candidate locus (r^2^ > 0.8). We also picked up rs12692878 as a proxy in a protective association on chromosome 2q24.3 because the RegulomeDB (https://regulomedb.org/regulome-search) showed a high probability that the SNP could influence transcription regulation^12^. The list of 8 candidate SNPs is shown in Table 2. Regional association plots for the 8 candidate SNPs are shown in Fig. 2 and Supplementary Fig. S2. All the 8 candidate variants were successfully genotyped, with a mean concordance rate of 99.9% (99.6–100%) in AESD cases.

**Table 2:** Candidate SNPs associated with AESD.

CHR	SNP	A1/A2	GWAS (252 cases vs. 792 controls)				Replication test (22 cases vs. 4773 controls)				Combined test (274 cases vs. 5565 controls)				Regulome DB^c^
			MAF in cases	MAF in controls	*p*^*a*^	OR (95%CI)	MAF in cases	MAF in controls	*p*-_Fisher_^b^	OR (95%CI)	MAF in cases	MAF in controls	*p*-_Fisher_^a^	OR (95%CI)	
2	**rs1850440**	T/C	0.44	0.31	2.44 × 10^–7^	1.71 (1.40–2.10)	0.39	0.36	7.56 × 10^–1^	1.10 (0.56–2.10)	0.43	0.36	3.11 × 10^–4^	1.38 (1.16–1.65)	1f.
5	**rs12656207**	G/C	0.39	0.27	1.49 × 10^–6^	1.67 (1.36–2.06)	0.31	0.28	6.09 × 10^–1^	1.17 (0.56–2.32)	0.38	0.28	3.66 × 10^–7^	1.60 (1.34–1.92)	NA
6	rs9349362	T/C	0.51	0.39	1.57 × 10^–6^	1.64 (1.34–2.00)	0.34	0.39	5.38 × 10^–1^	0.79 (0.40–1.53)	0.5	0.39	1.03 × 10^–6^	1.54 (1.29–1.83)	5
2	rs12616661	T/C	0.18	0.098	2.08 × 10^–6^	1.96 (1.48–2.60)	0.11	0.12	1.00	0.94 (0.29–2.38)	0.17	0.12	2.64 × 10^–4^	1.56 (1.22–1.96)	5
11	rs11213425	C/T	0.15	0.25	2.52 × 10^–6^	0.53 (0.40–0.69)	0.29	0.22	2.67 × 10^–1^	1.44 (0.67–2.90)	0.16	0.22	8.00 × 10^–4^	0.68 (0.53–0.86)	NA
19	**rs60651483**	T/C	0.16	0.26	4.19 × 10^–6^	0.54 (0.42–0.71)	0.18	0.22	7.14 × 10^–1^	0.80 (0.32–1.75)	0.16	0.22	5.92 × 10^–4^	0.67 (0.53–0.85)	5
18	rs7243486	T/G	0.16	0.26	4.46 × 10^–6^	0.54 (0.42–0.71)	0.31	0.23	2.72 × 10^–1^	1.47 (0.70–2.92)	0.17	0.24	3.47 × 10^–4^	0.67 (0.53–0.84)	4
2	rs12692878	T/C	0.38	0.49	7.57 × 10^–6^	0.63 (0.51–0.77)	0.48	0.45	7.57 × 10^–1^	1.12 (0.58–2.16)	0.38	0.45	1.51 × 10^–3^	0.75 (0.63–0.90)	1f.

The SNPs with replicable odds ratios in the replication study were shown in bold.

CHR: chromosome; A1: minor allele; A2: major allele; MAF: minor allele frequency; *p*-_Fisher_: *p*-value calculated using Fisher’s exact test; OR: odds ratio; 95% CI: 95% confidence interval; NA: not applicable.

^a^Genome-wide significance *p*-value = 5.0 × 10^–8^, suggestive significance *p*-value = 1.0 × 10^–5^.

^b^Significance levels were adjusted by the number of comparisons to correct for multiple testing. The significance level was set at *p* < 6.2 × 10^–3^ (adjusted α = 0.05/8).

^c^Functional prediction scores of each SNP by RegulomeDB database.

We also performed a replication analysis to further evaluate the 8 SNPs in 22 AESD patients who were not analyzed in the GWAS using TaqMan genotyping assays. The clinical characteristics of patients in the replication test are shown in Supplementary Table S1. Since the sample size was small, no correlations were observed in the replication analysis after applying the Bonferroni correction. However, the odds ratios of the 3 SNPs (rs1850440, rs12656207, and rs60651483) exhibited the same direction of association as the odds ratio of the GWAS (Table 2). Furthermore, combined analyses were performed using genotype data in the GWAS and replication study (Table 2). Only one SNP, rs12656207 reached the suggestive significance level (*p* = 3.66 × 10^−7^, odds ratio = 1.60).

### eQTL analysis

We selected the 3 SNPs, rs1850440, rs12656207, and rs60651483 for the cis-acting expression quantitative trait locus (eQTL) analysis because the odds ratio of these SNPs was replicated in the replication study. We then evaluated whether these SNPs affected the transcription of genes located near the variants using the Blood eQTL browser (https://genenetwork.nl/bloodeqtlbrowser/)^13^ and GTEx portal database V8 release (https://www.gtexportal.org/home/)^14^ (Supplementary Table S2 and S3). The top SNP, rs1850440 minor allele (T allele: disease-risk allele) correlated with the stronger expression of *STK39* in peripheral blood (*p* = 5.27 × 10^–11^, FDR < 0.05, Z-score = 6.56) from the Blood eQTL browser. No information was available for other tissues, including the brain, whereas GTEx showed the wide expression of *STK39* in the brain, including the cerebral cortex. Among the suggestive associations, rs12656207 was located 157-kb downstream of the F-box protein 38 gene (*FBXO38*) (Fig. 2b). Individuals carrying the G allele (i.e., the AESD-risk allele) of rs12656207 showed significantly higher expression levels of *FBXO38* in peripheral blood. The third variant, rs60651483, was located 20-kb upstream of the GIPC (GAIP interacting protein, C terminus) PDZ domain containing the family member 3 gene (*GIPC3*) (Fig. 2c). The relationship between *GIPC3* expression and rs60651483 genotypes in peripheral blood and tibial nerve was also detected in GTEx data; the *GIPC3* expression level of the rs60651483 minor allele (T allele: disease-protective allele) was significantly decreased in peripheral blood.

### In silico* functional analysis*

We evaluated the 65 SNPs with *p*-values < 1.0 × 10^−5^ in the GWAS to establish whether they are candidate functional variants that may influence transcription regulation using the RegulomeDB database and HaploReg v2 (https://pubs.broadinstitute.org/mammals/haploreg/haploreg_v2.php)^15^ (Table 2 and Supplementary Data). The top hit variant, rs1850440, showed a RegulomeDB score higher than 2a, suggesting its location in DNase hyper-sensitivity clusters and the binding of transcription factors. HaploReg showed the location of rs1850440 and SNPs in high LD (r^2^ >  = 0.6) with this SNP in enhancer histone marks, indicating that they regulate the expression of *STK39*.

### miRNA enrichment analysis

To identify candidate miRNAs associated with AESD, miRNA enrichment analysis was conducted using the GWAS data. The enrichment of pairwise association signals between miRNAs and their target genes was identified using our GWAS results of AESD (Table 3). Among these, the annotation confidence was high in miRBase for the 3 miRNAs: hsa-mir-34c, hsa-mir-449b, and hsa-mir-449c. Tissue-specific enrichment in the miRNA–target gene network of AESD was also detected for 8 different tissues (Supplementary Table S4). The anatomical category of each tissue included the lung, bone, immune system, and kidney.

**Table 3:** Candidate miRNA-target gene pairs associated with AESD.

miRNA	Annotation in miRBase	Genes	Gene description NCBI reference sequences
hsa-mir-1272	Insufficient data	*TNFRSF19*	The encoded protein is a member of the TNF-receptor superfamily
hsa-mir-34c	High	*GINS3*	The encoded protein is essential for the initiation of DNA replication and replisome progression
hsa-mir-4448	Insufficient data	*SCNM1*	*SCNM1* modifies phenotypic expression of *SCN8*A mutations
hsa-mir-449a	Insufficient data	*ASB4*	The protein encoded by this gene is a member of the ankyrin repeat and SOCS box-containing (ASB) family of proteins
		*GINS3*	
hsa-mir-449b	High	*ASB4*	
		*GINS3*	
hsa-mir-449c	High	*CLDN8*	The encoded protein plays roles in maintaining cell polarity and signal transductions
hsa-mir-4782	Insufficient data	*KCNIP1*	This gene encodes a member of the family of cytosolic voltage-gated potassium channel-interacting proteins
hsa-mir-623	Insufficient data	*KLHL20*	Members of this family are present throughout the cell and extracellularly with diverse activities

The enrichment of the pairwise association signals of miRNAs and their target genes was evaluated. The significance threshold of *p*-_Gene_ and *p*-_miRNA_ was α = 0.01.

## Discussion

Based on a clinical course of viral infection, fever, status epilepticus (early seizure), clustering focal seizures (late seizure), and cortical neuronal damage, AESD may be regarded as another syndrome of “acute encephalopathy with inflammation-mediated status epilepticus”^16^. Previous MR spectroscopic studies demonstrated the pathogenetic role of glutamate in cerebral cortical lesions after initial status epilepticus^17^. The appearance of characteristic lesions on MRI is as late as around the late seizure, delaying the diagnosis of AESD^18^. To enable an early diagnosis immediately after its onset, candidate biomarkers need to be identified.

Candidate gene analyses in Japan previously identified several susceptibility genes for AESD, such as common variants of carnitine palmitoyltransferase 2 (*CPT2*)^19,20^ and adenosine A_2A_ receptor (*ADORA2A*)^21^, and rare variants of the sodium voltage-gated channel alpha subunit 1 (*SCN1A*) and *SCN2A*^22,23^. However, AESD has not yet been studied using a genome-wide approach.

In the present study, GWAS for AESD patients identified 7 candidate loci reaching the genome-wide suggestive level. Among the 8 representative variants in these susceptibility loci, 3 SNPs, rs1850440, rs12656207, and rs60651483, showed odds ratios of the same direction between the GWAS and replication study. Regarding the first SNP, rs1850440, we found its location in the enhancer region of the *STK39* gene, and its regulation of *STK39* expression using the RegulomeDB database. cis-eQTL revealed a relationship between disease-risk allele T and the stronger expression of *STK39* in peripheral blood. *STK39* encodes a serine/threonine kinase mediating cellular stress-activated signals^24^. *STK39* is widely expressed in the brain, including the cerebral cortex in GTEx. In response to hypotonic stress with cell swelling, STK39 is activated and phosphorylates several cation–chloride cotransporters (CCCs). Based on the important roles of CCCs in the regulation of ion and water homeostasis in the mammalian brain, *STK39* has been implicated in cerebral edema^25^. STK39 activates the p38 mitogen-activated protein kinase (MAPK) pathway. A previous study reported that heat stress triggered the activation of p38 MAPK, leading to an increase in reactive oxygen species and the apoptosis of glial cells^26^. Proinflammatory cytokines, such as interleukin-1 and tumor necrosis factor-alpha (TNF-α), also activate the p38 MAPK pathway and induce cellular apoptosis. Status epilepticus up-regulates the expression of these cytokines in brain astrocytes and microglial cells^27^. Therefore, we speculate that the rs1850440-associated strong expression of *STK39* predisposes children to AESD because the onset of AESD is preceded by a high fever and status epilepticus.

Regarding the second disease risk SNP, rs12656207, our single-tissue eQTL analysis revealed disease-risk allele G correlated with higher expression levels of *FBXO38* in the blood. FBXO38, a ubiquitin ligase of programmed cell death 1(PD-1), is a negative regulator of T cell-mediated immunity^28,29^. The expression of PD-1 is up-regulated during acute viral infection^30^, a triggering factor of AESD. On the other hand, the third SNP, rs60651483, had a protective allele T for AESD. In the eQTL analysis, the T allele of rs60651483 correlated with the weaker expression of *GIPC3* in the blood. GIPC3, a PDZ domain protein, belongs to the GIPC family, which regulates a number of cellular processes, such as proliferation, planar cell polarity, cytokinesis, and migration^31^. Mutations in *GIPC3* have previously been reported in sensorineural hearing loss and audiogenic seizures^32^. The potential involvement of *FBXO38* and *GIPC3* in AESD warrants further study.

Using GWAS summary statistics, we conducted a miRNA enrichment analysis to identify miRNA and miRNA-target gene networks associated with AESD, which may provide additional insights into its pathogenesis as well as candidate biomarkers for an early diagnosis^8,33^. In the present study, we obtained 3 candidate miRNAs, hsa-mir-34c, hsa-mir-449b, and hsa-mir-449c, with high confident annotation in miRBase. These miRNAs belong to the mir-34/449 family, have similar sequences to each other, and are reportedly involved in immune responses and viral infections^34^. For example, hsa-mir-34c is expressed in human peripheral blood mononuclear cells following inflammation-associated endogenous damage^35^. Previous in vitro studies demonstrated that hsa-mir-34c derived from astrocyte exosomes exerted neuroprotective effects against cerebral ischemia–reperfusion injury by down-regulating the MAPK pathway^36^. On the other hand, hsa-mir-449b enhanced the activation of the interferon-β promoter induced by influenza A virus infection^37^. Therefore, we speculated that febrile status epilepticus caused by viral infection may provoke immune responses and up-regulate the expression of hsa-mir-34c and hsa-mir-449b, thereby inducing proinflammatory cytokines in AESD patients. The mir-34/449 family plays an essential role in the brain, especially in the development of forebrain, which is implicated in reward pathways, feeding, and social behaviors^38^. As the target gene of hsa-mir-449b, our miRNA analysis detected the *ASB4* gene encoding ankyrin repeat and suppressor of cytokine signaling box containing 4 (ASB4), which plays a role in proinflammatory responses up-regulated by TNF-α in endothelial cells^39^. The present results implicate these miRNAs of the mir-34/449 family, as well as the target gene *ASB4*, in the pathogenesis of AESD. Since they are all detectable in peripheral blood mononuclear cells, they have potential as biomarkers for the diagnosis of AESD.

There are several limitations in the present study. Firstly, due to the low incidence of AESD, the sample size was too small to find a locus of genome-wide significance and confirm reproducibility between the GWAS and replication study. The expected power for our GWAS was up to 42% at the genome-wide significant threshold under the additive model, assuming a genotype relative risk ranging between 1.7 and 2.0 and disease allele frequency of higher than 40%. GWAS achieved 84.3% to detect common alleles with a minor allele frequency ≥ 5%, genotype relative risk > 2.0, and disease allele frequency > 40% at a significant *p*-value threshold of 5.0 × 10^–8^ under the additive model when the number of cases was more than 450 (Supplementary Fig. S3). Secondly, the present study did not replicate previous findings on the susceptibility loci of AESD using a candidate gene approach (Supplementary Table S5)^19–21^. The reason for this discrepancy may be the small effect sizes of the variants reported previously and the insufficient sample size of the present study. Nevertheless, by using genome-wide approach, the present study revealed the pathogenetic roles of common genetic variants in AESD, a rare disease, as had previously been shown for other rare neurodevelopmental disorders formerly considered to be monogenic^40^. Thirdly, the present study could have detected variants and miRNA-target gene networks of febrile status epilepticus rather than those of AESD because most of the AESD cases have febrile status epilepticus at the onset. However, none of the SNPs and miRNAs found in this study have ever been described in previous studies on the genetic predisposition of febrile seizures. To directly address this question, another study using disease controls of febrile seizures is warranted.

In the present study, GWAS did not uncover definite susceptibility loci that contribute to AESD. Despite the limitations, we reported 3 variants with a suggestive association with AESD, including rs1850440 in the *STK39* gene. By integrating GWAS summary statistics and miRNA prediction software, we found the enrichment of GWAS signals on the networks of miRNAs and its target genes. These results may provide additional insights into the pathophysiology, earlier diagnosis, and better treatment of AESD.

## Materials and methods

### Collection of case samples and healthy control samples

Between 2008 and 2019, we recruited 254 Japanese pediatric cases of AESD. AESD was diagnosed in children with an acute onset of impaired consciousness after a preceding infection, meeting either or both of the following criteria: (1) febrile status epilepticus or biphasic seizures after the initial onset seizure, and (2) delayed appearance of the cerebral subcortical white matter lesions on cranial MRI^3^. In the GWAS, 418 healthy adults, residing in the Tokyo area and referred by the Genome Medical Science Project, the National Center for Global Health and Medicine (Tokyo, Japan), and 381 healthy adult controls from Pharma SNP Consortium (Tokyo, Japan) were recruited as healthy controls for this study. Genomic DNA was extracted from peripheral blood following a standard protocol. This study was reviewed and approved by the Institutional Review Board of the University of Tokyo. All methods were performed in accordance with the ethical guidelines and regulations. We obtained written informed consent from the parents of the patients and all participants.

### Genotyping of genome samples and QC in GWAS

**Figure 3:**
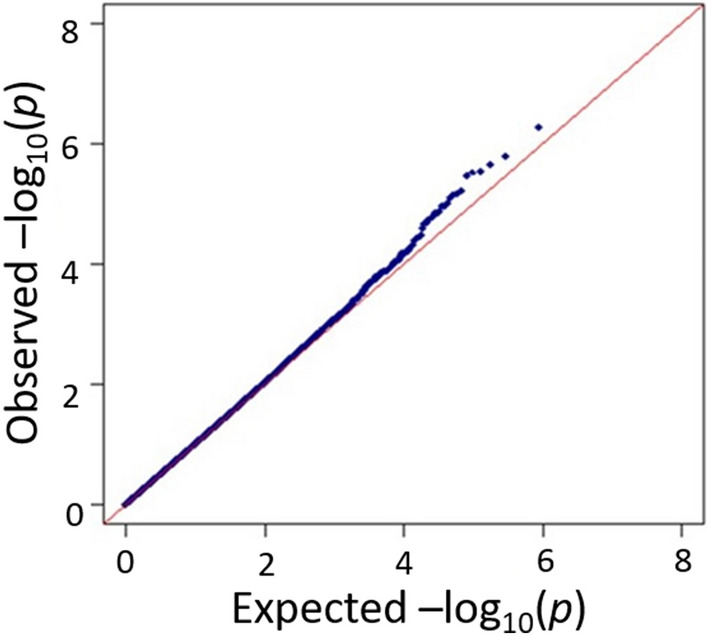
Quantile–quantile plot of AESD GWAS after whole-genome imputation. Quantile–quantile plot of *p*-values for each SNP calculated based on allelic model. The inflation factor was less than 1.004.

In the GWAS, SNPs were genotyped using the Affymetrix “Japonica Array v.2”^41^. UCSC hg19 was used as a reference genome. Genotype calling was conducted with the apt-probeset-genotype program in Affymetrix Power Tools ver. 1.18.2 (Thermo Fisher Scientific Inc., Waltham, MA). Sample QC was managed by following the developer’s recommendations: dish QC > 0.82 and sample call rate > 97%. We evaluated the clustering of each SNP using the Ps classification function in the SNPolisher package (version 1.5.2, Thermo Fisher Scientific Inc.). We used “recommended” SNPs allocated by the Ps classification function in subsequent analyses. Samples with overall call rates lower than 97% were excluded. The identity-by-descent (IBD) test was performed to detect cryptic relatedness. We eliminated subjects with PI_HAT values higher than 0.1875^42^. To eliminate population stratification, outliers in a principal component analysis (PCA) were also excluded. In the PCA, 97 JPT (Japanese in Tokyo, Japan), 106 CHB (Han Chinese in Beijing, China), 165 CEU (Utah residents with Northern and Western European ancestry), and 203 YRI (Yoruba in Ibadan), derived from HapMap phase III data, were used. We applied the following thresholds for genotyped SNP QC: SNPs were removed if they had a minor allele frequency (MAF) < 0.05, deviated from the Hardy–Weinberg equilibrium (HWE) *p* < 0.0001 in healthy controls, had SNP call rates < 99%, or were located in sex chromosomes or mitochondria. We collected 254 AESD cases and 799 healthy adult controls after genotyping the samples in the present study. In sample QC, 1 case and 3 controls were excluded by the IBD test. After PCA, 1 case and 4 controls were removed (Supplementary Fig. S4). The genomic inflation factor between cases and controls using a basic allele test after filtering was 1.004 (Fig. 3), suggesting that population stratification between selected cases and controls was negligible.

### Whole-genome imputation based on the 2KJPN panel

Pre-phasing was performed with EAGLE v2.3.242^43^. We conducted genotype imputation using IMPUTE4 v1.043^44^ with 2KJPN panel from a population cohort study performed by the Tohoku Medical Megabank Organization (ToMMo)^10,11^. After the whole-genome imputation, no sample was removed because of low call rate (< 97%). We excluded the same number of samples as the genotyped SNPs in the IBD test and PCA. QC criteria were the same as genotyped SNPs.

### Validation and replication test of candidate SNPs

For validation and replication studies, proxy SNPs were further selected to detect primary associations in each locus with suggestive significance (*p* < 1.0 × 10^−5^) applying either or both of the following criteria: a variant with the lowest *p*-value in the locus with strong LD (r^2^ > 0.8), and a variant with eQTL evidence in RegulomeDB v 2.0 (score > 2a)^12^. We performed a validation test using 8 candidate SNPs in the GWAS. SNPs were genotyped using TaqMan genotyping assays in the same 252 patients as in the GWAS set. To perform the replication study, we included 22 patients who were not analyzed in the GWAS, and also used TaqMan assays to genotype the cases. As controls in the replication test, we used information on allele frequencies in 4,773 samples from Integrative Japanese Genome Variation^10^.

### Statistical methods and software

In the GWAS, imputation analysis, and validation analysis, we calculated *p-*values using the chi-squared test in an allele frequency model. We used Fisher’s exact test for the replication and combined analysis. The Bonferroni correction was then performed by the standard method. We set the genome-wide significance level as *p* < 5.0 × 10^−8^ and suggestive significance level as *p* < 1.0 × 10^−5^. PLINK 1.9 was used for data cleaning and SNP-based analyses^45^. Manhattan plots and quantile–quantile plots were made using R software (version 3.6.2), and its package “qqman”^46^. Regional plots were generated using Locuszoom^47^. The statistical power of the current GWAS was calculated using the R package “CaTS”^48^.

### eQTL analysis

The relationship between the candidate SNP genotype and gene expression was examined using data available from the Blood eQTL browser (https://genenetwork.nl/bloodeqtlbrowser/)^13^ and GTEx portal database V8 release (https://www.gtexportal.org/home/)^14^.

### In silico functional analysis

We evaluated the functional probability of whether candidate variants influence transcription regulation using RegulomeDB database 2.0 (https://regulomedb.org/regulome-search)^12^ and HaploReg v2 (https://pubs.broadinstitute.org/mammals/haploreg/haploreg_v2.php)^15^.

### miRNA enrichment analysis

The enrichment of GWAS polygenic signals on miRNA–target gene networks was estimated using MIGWAS software^49^. The enrichment of the pairwise association signals of miRNAs and their target genes was quantitatively evaluated. The target genes of each miRNA were defined to have the top one percentile of target prediction scores in at least two prediction algorithms of MIGWAS software. Regarding each tissue with available miRNA expression data from the FANTOM5 consortium^50^, the cell type-specific enrichment of the GWAS signal in the miRNA–target gene network was evaluated using a permutation procedure^49^. We used miRBase (http://www.mirbase.org/)^51^ for the annotation confidence of the enrichment of the pairwise association signals of miRNAs. NCBI Reference Sequences (RefSeq) was used for the gene description (https://www.ncbi.nlm.nih.gov/refseq/)^52^.

## Supplementary Information

Supplementary Information 1.

Supplementary Information 2.

## Data Availability

The datasets generated and analyzed during the current study are available from the corresponding author on reasonable request.
