# Supplementary material for: GWAS identifies candidate susceptibility loci and microRNA biomarkers for acute encephalopathy with biphasic seizures and late reduced diffusion
Source: Sci Rep. 2022 Jan 25;12:1332. doi: 10.1038/s41598-021-04576-y (PMC8789807; doi:10.1038/s41598-021-04576-y)
Supplement: Supplementary file 2 — Supplementary Information 2. [file 41598_2021_4576_MOESM2_ESM.pdf]

GWAS identifies candidate susceptibility loci and microRNA biomarkers for acute encephalopathy with biphasic seizures and late reduced diffusion

Authors: Mariko Kasai * [1, 2], Yosuke Omae [3], Yosuke Kawai [3], Akiko Shibata [1, 2], Ai Hoshino [1], Masashi Mizuguchi [1], Katsushi Tokunaga [3]

Institutional affiliation:

[1] Department of Developmental Medical Sciences, Graduate School of Medicine, The University of Tokyo, Tokyo, Japan

[2] Department of Pediatrics, Graduate School of Medicine, The University of Tokyo, Tokyo, Japan

[3] Genome Medical Science Project, National Center for Global Health and Medicine, Tokyo, Japan

Correspondence:

Mariko Kasai

kasaim-tky@umin.ac.jp

Supplementary Table S1. Clinical characteristics of patients in the replication study

**Clinical characteristics**	**Number of the patients**	
	**N=22**	**%**
**Sex**		
Male	12	54.5
Female	10	45.5
**Age (months)**		
<12	2	9
12-24	9	40.9
>24	11	50
**Early seizures** [a]		
Status epilepticus (>15min)	19	86.4
Short seizure (<15min)	3	13.6
**Late seizures** [b]		
Yes	11	50
No	11	50
**MRI findings** [c]		
Yes	16	72.7
No	6	27.3
**Outcome**		
Full recovery	6	27.3
Neurological sequelae [d]	14	63.6
Death	0	0
Unknown	2	9

[a] Early seizures: generalized convulsion in the early phase of AESD, [b] Late seizures: cluster of focal seizures in the late phase. [c] MRI findings: characteristic lesions in the cerebral subcortical white matter detected by cranial MRI. [d] Neurological sequelae: intellectual and/or motor disability.

Supplementary Table S2. Peripheral blood eQTLs for susceptibility variants

**SNP**	**Gene**	***p*-Value**	**FDR**	**Alt**	**Ref**	**Z score**
rs1850440	*STK39*	5.3×10^{-11}	0.00	T	C	6.56
rs12656207	*FBXO38*	5.1×10^{-17}	0.00	C	G	-8.39
rs60651483	NA	NA	NA	NA	NA	NA

eQTL analyses of susceptibility variants were conducted based on the Blood eQTL browser. The Z score of each SNP was for the alternative allele in peripheral blood. Abbreviations: Alt: alternative allele; Ref: reference allele; NA: not available.

Supplementary Table S3. Single-tissue eQTLs for susceptibility variants

SNP	Gene	*p*-value	Alt	Ref	Effect size	Tissue
rs1850440	NA	NA	NA	NA	NA	NA
rs12656207	*SPINK6*	3.2×10^{-11}	G	C	0.32	Esophagus - Mucosa
rs12656207	*SPINK13*	1.2×10^{-7}	G	C	-0.29	Thyroid
rs12656207	*SPINK6*	6.1×10^{-7}	G	C	0.27	Lung
rs12656207	*FBXO38*	4.0×10^{-5}	G	C	0.061	Whole Blood
rs60651483	*GIPC3*	9.4×10^{-7}	T	C	-0.48	Nerve - Tibial
rs60651483	*GIPC3*	1.8×10^{-5}	T	C	-0.35	Whole Blood
rs60651483	*TBXA2R*	5.9×10^{-5}	T	C	-0.33	Nerve - Tibial

eQTL analyses of susceptibility variants were conducted based on the GTEx portal database. The effect size of each SNP was for the alternative allele in every organ. Abbreviations: Alt: alternative allele; Ref: reference allele; NA: not available.

Supplementary Table S4. Cell type-specific enrichment of GWAS signals in the miRNA–target gene network with AESD

Tissues	Anatomical category	*p*-value	Fold change
Smooth muscle cell of the pulmonary artery	Lung	0.019	2.94
Human chondrocyte de-differentiated sample	Bone	0.02	2.78
Mesenchymal stem cell of the bone marrow	Immune	0.024	4.4
Nephron tubule epithelial cell	Kidney	0.025	2.7
Epithelial cell of a nephron	Kidney	0.027	2.68
Human chondrocyte re-differentiated sample	Bone	0.042	2.27
Nucleus pulposus cell of an intervertebral disc	Bone	0.046	3.71
Immature conventional dendritic cell	Immune	0.049	2.47

The tissue category of each cell was used according to the annotation of developers. Enrichment *p*-values of miRNA-target gene network signals 0.05 or lower were considered to be significant, which developers adopted.

Supplementary Table S5. Susceptibility variants previously reported to be associated with AESD

Chr	Pos	SNP	Gene	Minor	Major	OR (95%CI)
1	53676401	rs2229291	*CPT2*	G	T	1.67 (1.06-2.69)
22	24825511	rs2298383	*ADORA2A*	C	T	1.70 (1.17–2.45)

The odds ratio was reported in previous studies using a candidate gene approach.

Abbreviations: Chr: chromosome; Pos: base pair position; Minor: minor allele; Major: major allele; OR: odds ratio; 95%CI: 95% confidence interval; *CPT2*: carnitine palmitoyl transferase 2 gene; *ADORA2A*: adenosine A2A receptor gene.

Supplementary Figure S1. Regional plot of GWAS after conditioning on rs1850440

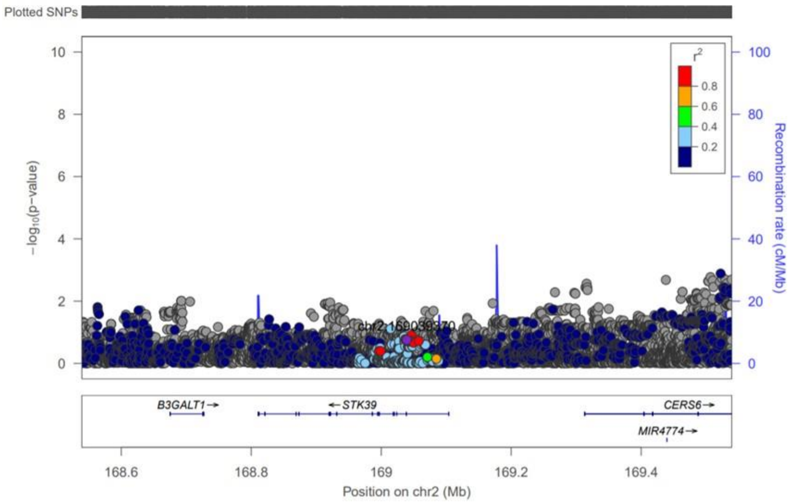

There was no secondary signal in the 2q24.3 region.

Supplementary Figure S2. Regional plots of candidate variants selected for the validation and replication study

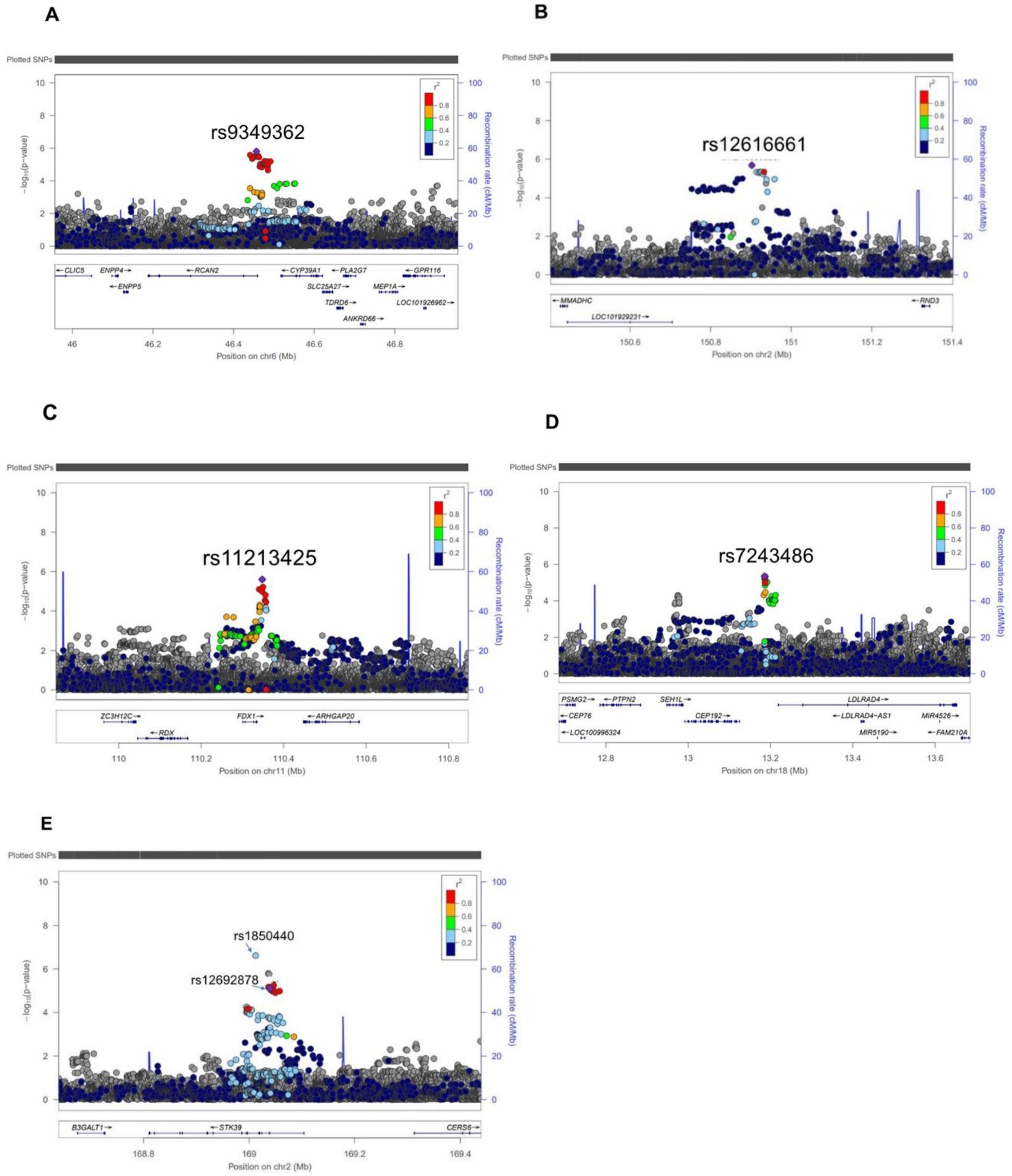

Regional plots of susceptibility variants other than rs1850440, rs12656207, and rs60651483 based on the association analysis in the GWAS. The purple dots indicate the focused variants.

Supplementary Figure S3. Statistical power of the current GWAS

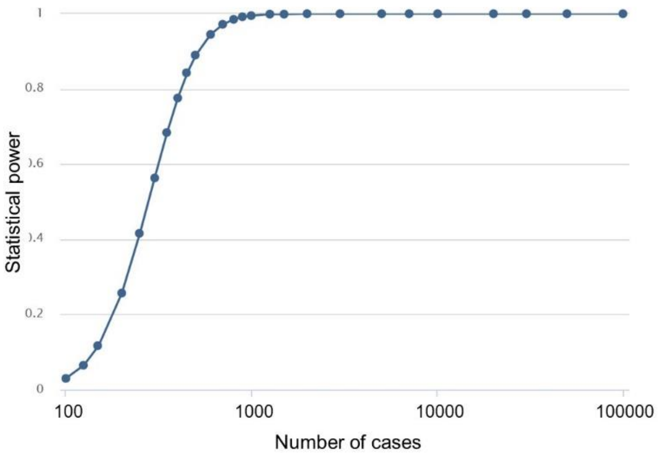

The dots show the statistical power of our GWAS when we assumed a disease prevalence of 0.01% in a Japanese population under an additive model for variants with an allele frequency of 5% using 254 cases and 799 controls. The significance threshold *p*-value was 5.0×10^{-8}. When the number of cases was more than 450, the study power was 84% to detect common alleles with a minor allele frequency ≥ 5%, genotype relative risk > 2.0, and disease allele frequency > 40%.

Supplementary Figure S4. Results of a principal component analysis (PCA) of samples and reference populations

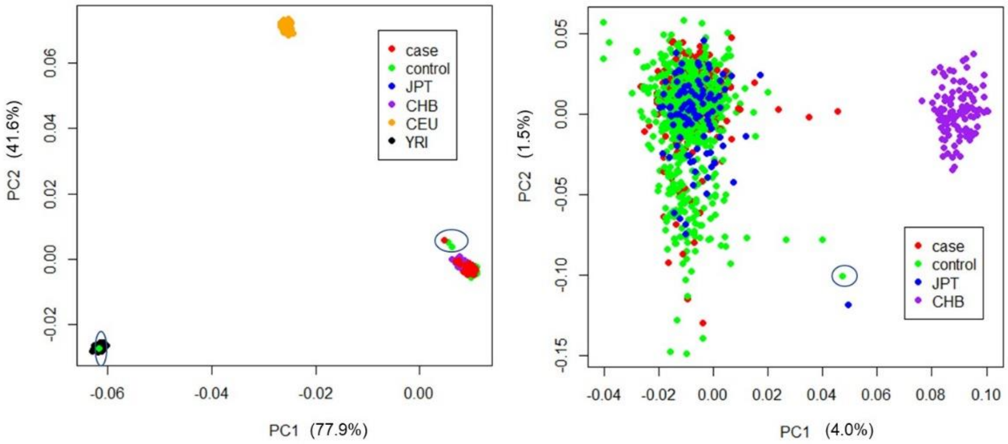

In the PCA, 97 JPT, 106 CHB, 165 CEU, and 203 YRI were used as references that were derived from HapMap project phase 3. PCA found 5 outliers, 1 case and 4 controls, to be excluded. They were defined visually. The outliers were highlighted by circles.

Abbreviations: PC1: principal component 1; PC2: principal component 2.

Abbreviations: JPT: Japanese in Tokyo, Japan; CHB: Han Chinese in Beijing, China; CEU: Utah residents with Northern and Western European ancestry; YRI: Yoruba in Ibadan.
